# Has Rural-Urban Migration Promoted the Health of Chinese Migrant Workers?

**DOI:** 10.3390/ijerph17041218

**Published:** 2020-02-13

**Authors:** Cuihong Long, Jiajun Han, Yong Liu

**Affiliations:** 1 School of Economics, East China Normal University, Shanghai 200062, China; chlong@jjx.ecnu.edu.cn (C.L.); 51194401011@stu.ecnu.edu.cn (J.H.); 2 School of Economics, Sichuan University, Chengdu 610065, China

**Keywords:** migration, health, urbanization, China

## Abstract

The relationship between health and migration has always been an important theme in immigration research. This research develops a new approach to test the healthy migrant hypothesis and the salmon bias hypothesis in China by examining an interaction term combining agricultural hukou and migrant status, non-agricultural employment history, and subsequent area of residence. Based on two Chinese micro-databases, CGSS 2015 and Harmonized CHARLS, we conducted an empirical test on the relationship between migration and health. Our empirical evidence suggests that the initial health advantage among Chinese rural migrant workers was largely due to self-selection rather than migration effects. After controlling for demographic and socioeconomic characteristics, this advantage disappeared. After their health deteriorated, migrant workers returned to their original location. This could exacerbate the contradiction between the allocation of medical resources and the demand in rural and urban China, further intensifying the already widening health status gap between rural and urban residents.

## 1. Introduction

Population migration, including domestic and international migration, is an objective phenomenon in the process of globalization and urbanization. China is the most populous and largest developing country. Since the 1970s, with the advancement of reform and opening up, China has experienced a rapid urbanization process that has lasted more than 40 years. It has also witnessed the largest domestic migration in the world, with hundreds of millions of people moving from rural to urban areas. This migration has had a profound impact on the economic and social development of China and the world. The relationship between migration and health is a topic that cannot be avoided in international migration research. Existing research on international migration has usually focused on two well-known hypotheses. The first is the healthy migrant hypothesis, which holds that migrants represent a previously selected group composed of individuals whose initial health condition is better than others due to the highly demanding, challenging, and stressful migration process and the adaptability required to live in destinations [1,2,3,4,5,6]. The second is the salmon bias hypothesis, which holds that elderly migrants who experience health deterioration have a higher propensity to return to their hometown rather than remaining in destination cities [6,7,8,9,10].

The relationship between migration and health is also important for domestic migration studies. Rural-urban migration is an important and irreplaceable channel for rural migrants to achieve the optimal allocation of human capital and cast off the chains of poverty in developing countries [11]. Considering that the general human capital and special human capital of rural migrants are both relatively vulnerable [12,13], better health conditions are especially important to Chinese rural migrant workers. These workers are the backbone of China’s urban construction; therefore, their health deserves constant attention. On the one hand, there are many commonalities between China’s internal migration and international migration [14,15,16,17]. On the other hand, given the complex set of conditions produced by the Chinese household registration system (commonly called the hukou system) that affect rural migrant workers, China’s internal migration demonstrates its own particularity and complexity. Consequently, further verification is required to observe whether the aforementioned two hypotheses regarding migration and health are tenable for China’s urban-rural migration.

This article is based on two recently available Chinese micro-databases, the 2015 Chinese General Social Survey (CGSS 2015) and the Harmonized China Health and Retirement Longitudinal Survey (Harmonized CHARLS), which provide empirical data on the relationship between migration and health. Our empirical results buttress both the “healthy migrant hypothesis” and the “salmon bias hypothesis” in the Chinese context. The rest of this article is organized as follows: Section 2 discusses the literature related to migrants and health and presents the empirical strategies. Section 3 demonstrates the empirical results and Section 4 concludes.

## 2. Materials and Methods

### 2.1. Literature Review

We focus on the most populous and the largest developing country, China, and examine the literature on the health conditions of the protagonists of domestic migration (i.e., Chinese rural migrant workers or peasant workers), movement, and their relation. Bryan et al. [11] note that large cities have been irreplaceable conduits from destitution to affluence in the underdeveloped world, and rapid urbanization inevitably involves a massive scale of internal rural-to-urban migration. Conclusions regarding the health effects of internal migration on Chinese rural migrant workers are equivocal and multifaceted in the existing literature.

Akram et al. [18] note that China’s hukou system, which restricts freedom of demographic dynamics, is the most important contemporary example of mobility confinement. Similarly, Li and Rose [19] illustrated multifarious dimensions of urban social exclusion resulting from hukou. Qian et al. [20] found that under this restrictive system, migrants without long-term plans to reside in host cities have a lower tendency to establish their health records. Given the important role that health records play in managing health, facilitating patient-physician communication, and ensuring continuity of care, the absence of health records could lead to negative health consequences. Lu et al. [21] and Jianlin Niu [22] identified adverse effects of internal migration on peasant workers’ health. Li et al. [23] noted that long working hours, which are common among rural migrant workers, could explain health deterioration. Lin et al. [24] underlined the association between social integration, income equality, and health status. Li Ji [25] concluded that health status worsened with greater urbanization. Qiu et al. [26] discovered a high prevalence of depression symptoms among migrant workers within Sichuan Province. With regard to the circumstances of provinces, Qin et al. [27] also found that the negative effect on health produced by migration outweighed the positive effect, and the negative health effect on female migrant workers was even more complex. However, no gender association was found in migrant workers in the research of Mou et al. [28]. The results from Song and Sun [29] demonstrate some differences in the effects of migration on health. These authors divide internal migration into two categories, short-term and long-term migration, and suggest that short-term migration has a positive effect on health due to better payment in inflow areas, although in the long run, the positive effect becomes insignificant. In accordance with Song and Sun [29], Tong and Piotrowski [15] found that in 1997 and 2000, respondents who reported “excellent” self-reported health were more likely to be migrant workers; however, in 2004 and 2006, this phenomenon disappeared among the same group of respondents. These authors also indicated that family accompaniment could buffer deleterious health effects to a certain extent. Chen and Zhang [30] demonstrated that the tendency towards migration with families has increased; unfortunately, the restrictive hukou system limits this buffering effect. Notwithstanding the potential health deprivation in cities, Mou et al. [31] indicated that return to the countryside could exacerbate the difficulties of life, especially for female peasant workers. Other research has compared the health status of rural migrant workers and other groups. For instance, Lu and Qin [32] emphasized that the degree of health selectivity is stronger in China than in other developing countries, and rural residents with better health are more likely to migrate.

For those left behind by migrant workers, Lu et al. [33] found that receipt remittances could partly compensate for the deficiency of family company. These results are in accordance with those of Xiao et al. [34], who identified three sources of pressure: Parents’ insufficient pensions, children’s education expenses, and medical expenses. However, Zhang et al. [35] revealed that the physical health status of migrant workers is significantly better than that of rural residents but cannot compete with urban residents’ health. Similarly, Lu and Qin [32] found that compared to native citizens in host cities, migrant workers do not exhibit health advantages. The conclusions of Yi and Qi [36] are consistent with ours: The health conditions among rural migrant workers is better than that of native citizens because better health is a prerequisite of migration. Despite the different conclusions among existing studies, all of these studies support the “healthy migrant hypothesis” and the “salmon bias hypothesis”, to some degree.

With respect to mental health, Dai et al. [37] concluded that rural migrant workers had a lower risk of depression than their peers who remained in rural areas, although these results were preliminary. Shang et al. [38] noted that working outside of their hometown could increase the tendency towards depression among rural migrant workers, and returning to their hometown consistently produced worse health conditions. Yi et al. [39] concluded that peasant workers have the worst emotional health. Wang et al. [40] suggested that more perceived stigma and discrimination in host cities leads to disillusionment, which could further lead to higher levels of psychological distress. In contrast, Juan Chen [41] focused on rural migrant workers in Beijing and found that although the initial physical health advantage gradually vanished, mental health improved as they became more settled in Beijing. Qi et al. [17] did not discover any psychological disparities among different groups with regard to physical health, and after controlling for major demographic and socioeconomic characteristics, many physical health indicators no longer exhibited significant differences. Qi and Niu [16] also found that most of the health disparities disappeared after standardizing demographic and socioeconomic characteristics. Niu and Qi [42] found that long working hours, a poor working environment, and barriers to achieving health services in inflow areas all contribute to health deprivation among rural migrant workers. Intuitively, rural migrant workers’ poor living conditions could also decrease their health advantage. However, an article by Li and Liu [43] indicated that although the dormitories provided by employers are often dilapidated, dormitory tenants possess the best mental well-being and perceive the least stress compared to other housing dwellers. The authors believe that employer-provided dormitories could alleviate the tenants’ pecuniary burden, which further mitigates the negative effect of migration. Other perspectives can shed light on the mechanism of health divestiture among rural migrant workers. For instance, Anning Hu [13] underscored the impact of education. Rural residents must be educated longer to manifest an educational health promotion effect given the inferior quality of China’s rural education; rural migrant workers are often worse educated and therefore lack this channel to overcome many barriers in working areas. Ling Zhu [44] found a new angle by emphasizing the administrative aspect: The assessment of Chinese officials’ performance involves competing in attracting businesses and inviting investments. Thus, the local government is biased towards employers’ benefits and ignores appeals from employees. Administration-oriented labor protection is often at variance with the operation of a market economy, which could further debilitate rural migrant workers’ health. Despite the initial health advantage they possess, they cannot maintain their health under this official incentive mechanism. Summerskill et al. [45] emphasized that as migrants compose more than 15% of the urban population in China, the systematic segregation caused by hukou is contrary to good public health practice. They also noted that the disparities among China’s three public health insurance schemes provide financial protection disproportionately in favor of native urban citizens. Consequently, outpatient departments in tertiary urban hospitals are overloaded with patients whose basic medical requirements are often mismatched to the high-level expertise available. The regulations of the hukou system and the distortion of primary care may lead to the misallocation of medical resources.

Although some existing literature on the health of Chinese migrant workers partially validates the “healthy migration hypothesis” or the “salmon bias hypothesis”, the source of the initial health advantages shown by migrant workers remains to be further explored. The existing literature does not reveal the process of the health deterioration of migrant workers. This article improves existing research methods by optimizing the factor analysis approach and employing Propensity Score Matching-Difference in Difference (PSM-DID) and other techniques to more precisely analyze the source of migrant workers’ health advantages and the process of health depletion, to better understand the trajectory of migrant workers’ health status and extend similar research. In addition, we use the mixed-effect logit regression (also called hierarchical logit) model to allow the random intercept and random slope to vary with the sample survey region, thereby enhancing the reliability and universality of the research results.

### 2.2. Empirical Strategy

#### 2.2.1. Data Source

The data used in this article are the 2015 Chinese General Social Survey (CGSS 2015) and the Harmonized China Health and Retirement Longitudinal Survey (Harmonized CHARLS), both of which are nationally representative micro-databases. A particular advantage of choosing the CGSS2015 to investigate the healthy migrant hypothesis is that this survey is nationally representative. It was jointly conducted by Renmin University of China and academic institutions across the country. This survey involved a sample of 10,968 households in 28 provinces, autonomous regions, and municipalities directly under the central government (excluding Hong Kong, Macao, and Taiwan). Considering that seeking jobs in cities always precedes returning to their hometown, we first tested the healthy migrant hypothesis in China using the CGSS2015. Table 1 presents the descriptive statistics regarding the variables from CGSS 2015 used in this paper.

As part of the robustness check, we also applied the Harmonized CHARLS to examine the salmon bias hypothesis in China. Given that most of the respondents included in the Harmonized CHARLS were older than 45 years, members of this age group whose hukou origin was ascribed to the agricultural were more likely to experience working outside their hometown. There are few existing articles using the CGSS data set for factor analysis. Due to simplification, many related variables that may influence health conditions are combined into a few potential dimensions in this paper. When practicing factor analysis, the principal factor method of iterative common factor variance is used. This iterated principal-factor method can obtain the best explanatory potential dimension of the relevant model among numerous variables by re-estimating the communalities iteratively, which is an ameliorating approach based on the principal component factor method used in early documents. During this process of generating factors, we chose oblique rotation instead of the orthogonal rotation used in existing articles, which allows overlapping variance among variables. The same factor analysis approach was employed again in the Harmonized CHARLS data set to generate different factors representing different dimensions of health.

#### 2.2.2. Descriptive Statistics

The CHARLS National Statistical Survey was implemented in 2011 and covers 150 county-level units, 450 village-level units, and 17,000 individuals in approximately 10,000 households. These samples are tracked every two or three years. The particular advantage of using the Harmonized CHARLS to investigate the health effect of mobility is that it contains copious amounts of indicators of health condition measurements and focuses on China’s population aged 45 and older. Members of this age group with agricultural hukou are more likely to experience working outside their hometown. We provide descriptive statistics on the selected variables in the Harmonized CHARLS shown in Table 2.

#### 2.2.3. Model Selection

We are interested in the impact of internal migration on health conditions among Chinese peasant workers. We used the sequencing variable self-rated health (SRH) to define the health status, where 1 to 5 indicates very unhealthy to very healthy, respectively. As mentioned above, since the Chinese household registration system leads to rural-urban dual demarcation, the differential possession of hukou categories allows us to employ the difference-in-difference technique to capture the causal effect of internal migration on health status among different categories of hukou owners. Let mobility be a person’s migration status. If “current hukou registration place” is “outside the district/county level”, then our critical independent variable mobility equals 1; otherwise, it equals 0. Let *agrihk* be a person’s hukou indicator, equaling 1 if “current hukou registration status” is “agricultural hukou” and otherwise equaling 0. We obtain four groups as shown in Table 3.

We use the DID (Difference-in-Difference) model to control for systematic differences among both the migrant status group and the hukou category group.

(1) $$SRH_{i}=\alpha_{0}+\alpha_{1}mobility_{i}+\alpha_{2}agrihk_{i}+\alpha_{3}mobility_{i}\times agrihk_{i}+\varepsilon_{i},$$

The hukou invariant and migrant status-specific elements were eliminated in E(SRH|*mobility* = 1, *agrihk* = 1)-E(SRH|*mobility* = 1, *agrihk* = 0) and E(SRH|*mobility* = 1, *agrihk* = 1)-E(SRH|*mobility* = 0, *agrihk* = 1), respectively, while other changes not from migrant status could be eliminated through the difference of $\Delta E\left(SRH_{i}|mobility=1\right)-\Delta E\left(SRH_{i}|mobility=0\right)$. Put differently, other related factors may affect health, which could be removed through the DID technique (Table 4).

Our interest is in the coefficient of the interaction term, which is the DID (Difference-in-Difference) estimator. As shown in Table 4, it is significantly positive. Is this because migration has improved the health of migrant workers? However, the DID technique requires a consistent pre-trend between the treated group and the control group given that better health conditions are required to overcome many kinds of barriers to working in destination cities, especially in China, where the restrictive hukou system makes health more important for migrants. We need to control for other demographic, domestic, and socioeconomic characteristics to determine whether these factors differentiate between agricultural hukou and non-agricultural hukou citizens when mobility equals zero.

(2) $$SRH_{i}=\alpha_{0}+\alpha_{1}mobility_{i}+\alpha_{2}agrihk_{i}+\alpha_{3}mobility_{i}\times agrihk_{i}+X_{i}\beta +\varepsilon_{i}$$

where *X_i_* is a vector of control variables that includes age, gender, and marriage status.

## 3. Results

Table 5 presents the results from the DID estimation. Although the coefficient of the DID estimator still remains positive and statistically significant at the 5% level, the decline of its absolute value indicates that demographic characteristics play an important role in explicating the health condition. According to the results of the *t*-test, young and single individuals may tend to move to cities in hopes of better economic opportunity. Generally, young peoples’ health status is better than that of elderly people, therefore, the health advantage could partly attribute to the youth and vigor; the empirical results are parallel with previous studies [6,16,17,22,36,42].

Given the heterogeneity of the pre-trend between the treated and control groups according to the result of the *t*-test, we employed propensity score matching to mitigate the selection bias and obtain a more comparable treatment and control group. Finally, we combined the PSM and DID to find a more reliable average health effect of migration on Chinese peasant workers.

In the first step, we used the logit regression to estimate the propensity score of entering the treatment group (migrant). In addition to age, gender, education level, and marital status, we want to add socioeconomic characteristics as covariates to match, thereby further enhancing the comparability of the control group.

Considering the conciseness of the regression framework, we decided to use factor analysis according to Hamilton [46]. The principal factor method using iterated communalities can identify the potential dimension that best explains the correlation among the variables. We utilized the principal factor method of iterative common factor variance and then applied factor oblique rotation, which simplifies the factor pattern and allows some degree of correlation among factors. Although related factors are less parsimonious in statistical significance because they have overlapping variances, if the factors generated in this article are considered to be dimensions that reflect socioeconomic status and are not necessarily irrelevant, the use of oblique rotation is more in line with the actual situation than the orthogonal rotation commonly used in the existing literature. According to the KMO (Kaiser-Meyer-Olkin) test result shown in Table 6, the KMO value of the majority of our selected variables is more than 0.7 and the overall KMO value is 0.6775, which implies that factor analysis is appropriate here.

According to the loading of different variables on factors, we extracted three factors: Subjective socioeconomic perception, career influence, and objective socioeconomic status. The subjective socioeconomic perception factor includes the self-rated family financial situation, socioeconomic status compared with peers, autonomy in determining the way of working, frequency of depression, social equality perception, and whether a CCP member or not. The career influence factor includes whether the company is within the system and whether the company is a state-owned enterprise. The objective socioeconomic status factor includes household income per capita, whether the respondent possesses a private car, whether the respondent has medical insurance, whether the respondent signed a written labor contract with the current employer, the respondent’s fluency level of speaking Mandarin, and the frequency of participation in cultural events.

**Figure A1:**
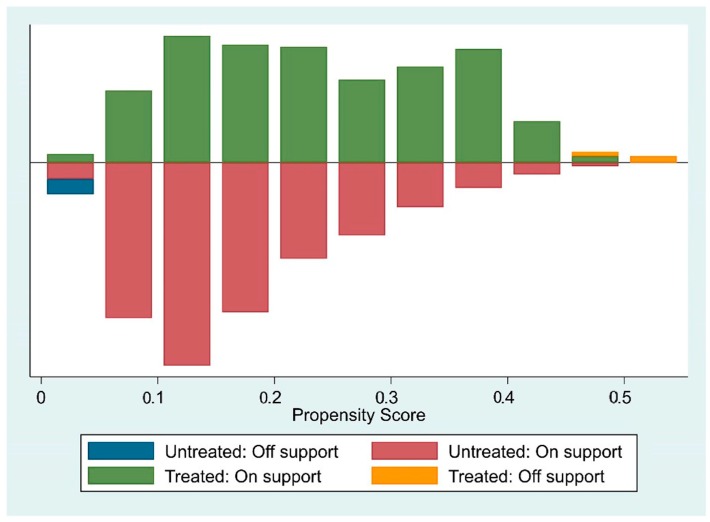
Standardized bias of each variable.

**Figure A2:**
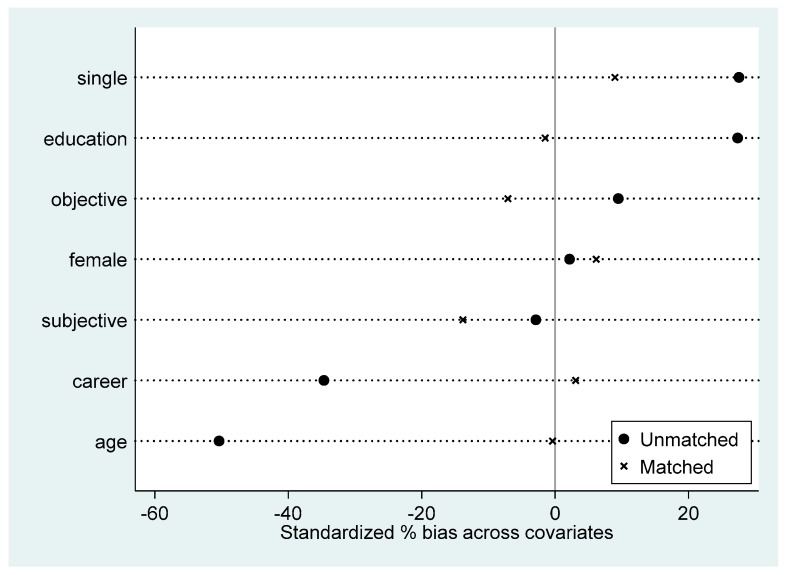
Standardized bias of each variable.

After completing the factor construction, we sorted the data randomly and subsequently implemented the PSM and PSM-DID approaches. A one-to-one match was primarily used (using the closest propensity score observed as a control). Kernel matching and local linear regression matching were performed for the robustness test. We first used the one-to-one match, as shown in Appendix A: Figure A1. Most of the variables’ standardized bias narrowed after matching. According to Appendix B: Figure A2, the majority of the observed variables were in the “On Support” category, which indicates only slight loss of samples during PSM. We subsequently constructed two other matching techniques for robustness checks, kernel matching, and local linear regression matching. As shown in Table 7, there were no conspicuous differences among the different PSM approaches, which implies that the PSM approach does not depend on the specific method.

In the next step, we used the PSM-DID (Propensity score matching combined Difference-in-Difference) model to construct a more comparable control group. As shown in Table 8, the DID estimator is neutralized after the PSM-DID process, which indicates that the migrant workers’ health priorities could disappear after controlling their attributes.

The balanced test shown in Table 9 indicates that the PSM approach played a role in eliminating the differences between the two groups, the mean value of selected covariates between treatment group and control group didn’t demonstrate significant difference anymore, which suggest that practicing PSM-DID is apposite here.

Given that our dependent variable of health status is defined by self-rated health, which is the ordinal variable, we decided to employ ordered logit regression and mixed effect logit regression to conduct a robustness check.

First, we used the ordered logit approach. As shown in Table 10, in Model 1, we included only migrant status, hukou status, and their interactive term. In Model 2, based on Model 1, we added further demographic variables, such as age, gender, marital status, and the interaction term combining gender and marital status. In Model 3, based on Model 2, we added education level. In Model 4, Model 5, and Model 6, we added three previously generated factors: Subjective socioeconomic status, career influence, and objective socioeconomic status. Finally, in Model 7, we added three factors simultaneously based on Model 3.

According to Model 1, we incorporated only migrant status, hukou category, and their interaction term. The coefficient of mobility was positive and significant at the 1‰ level, and the coefficient of agricultural hukou was negative and significant at the 1‰ level, conforming to common sense. The migration progress is highly demanding on health. The widening gap between rural and urban China, including but not limited to basic infrastructure, sanitary conditions, public goods, medical care services, and the affordability of remedies, could lead to deleterious effects on rural residents’ health [13,22,36]. In addition, farming may involve pesticides, which could also harm farmers. However, the coefficient of our interaction term is positive and statistically significant at the 1‰ level and possesses the highest absolute value, which accounts for the healthy migrant hypothesis, the stressful migrant process and working in unfamiliar cities. Thus, better health conditions are a prerequisite. Furthermore, in rural China, the quality of education lags far behind that of cities, which causes lower general human capital [47] among rural migrants. Their urban employers do not want to invest in job training for them because the hukou system, such as the Chinese internal passport, inhibits rural migrant workers from settling in the destination city; therefore, the paucity of job training reduces their specific human capital. This lack of human capital makes health particularly valuable to the migrant workers. In Model 2, when we add age, gender, and marital status based on Model 1, the coefficient of mobility declines in both absolute value and statistical significance, as did the interaction term of mobility multiplied by agricultural hukou. The coefficient of age is negative and significant at the 1‰ level, which is normal since young people are generally healthier than the elderly. According to our results, females and single women tend to be less healthy. Interestingly, the interaction term combining females and single women is positive and significant at the 5% level, which may suggest that as Chinese rural women move to cities, they can disentangle themselves from violent husbands and overbearing in-laws, women in rural China are more likely to suffer domestic violence than female citizens in urban China [48,49,50]. Due to urbanization, the grip of tradition loosens, and women possess more choices about whom they marry or live with; therefore, living among strangers in metropolises may not be a cause for despair but a chance to throw off the fetters of custom and kinship. All of these causes make their lives more bearable and lead to better health status among single women. In Model 3, when we add education, the coefficient of mobility becomes zero, and the coefficient of the interaction term combining mobility and agricultural hukou also declines compared to Model 1. The protective effect from education to health is revealed in Model 3. As shown in the regression results of Models 4 to 7, we discovered that the statistical significance of mobility, agricultural hukou, and their interaction term decreased as other demographic, cognitive, and socioeconomic characteristics were gradually incorporated in the model, and the coefficient of mobility turned from positive to negative. Both the absolute value and the statistical significance decreased in the coefficient of the interaction term combining mobility and agricultural hukou, which implies that migration does not have a positive effect on health in China. The initial positive and significant health effect could be the result of self-selection since healthier individuals are more capable of migrating. We note that the preponderance of rural migrant workers cannot obtain necessary medical treatment because of the lack of a local hukou, which determines their access to public health services in the destination cities. When a dangerous work environment and dilapidated residences lead to a precipitous deterioration in migrant workers’ health and increase their demand for medical treatment, they are more likely to return to their hometown to address their declining health. The coefficient of the interaction term combining single and female and the coefficient of education becomes insignificant but also positive. The advantages of being single for women and education may be reflected in socioeconomic factors.

However, people in different regions may have different criteria about their health status. In the next step, we divide self-reported health into dual dummy variables, with “very unhealthy”, “less healthy”, and “ordinary” equaling zero and “fairly healthy” and “very healthy” equaling one. We employ mixed-effect logit model to allow the intercepts and slopes to vary among respondents from different regions. First, we include each place of the interview as a random intercept in the mixed-effect logit model. In the regression result, compared to the normal logit regression, the likelihood ratio test indicates that the random intercept manifests significant disparities. We reconsider the seven aforementioned models using mixed-effect logit regression, as shown in Table 11. When we incorporate the random intercept of every interview location into the mixed-effect logit model, we can observe that the positive effect from mobility to rural migrant health decreases as other demographic, cognitive, and socioeconomic factors are gradually brought into the function, which corresponds to our assumption. The initial better health condition was largely due to self-selection given that better health could be rural migrant workers’ most important competitive advantage.

**Figure 1:**
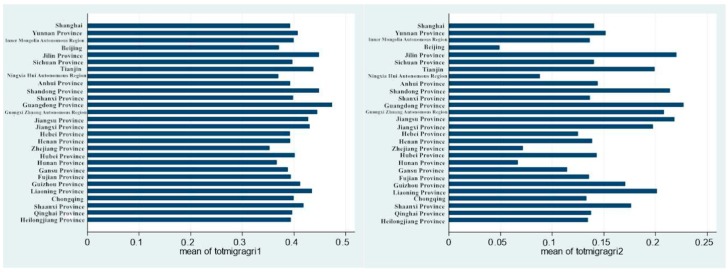

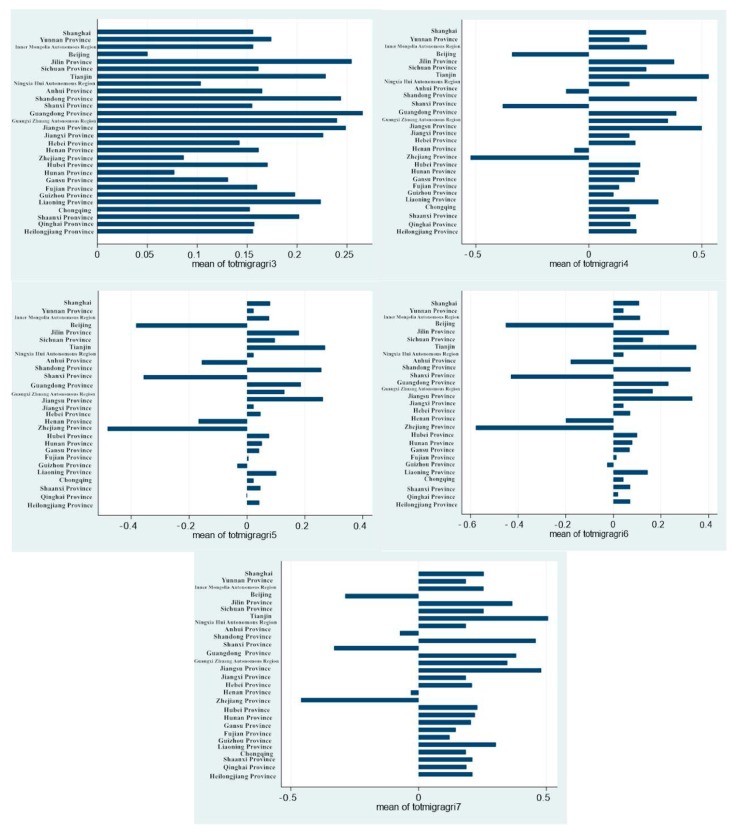
The total effect (= random effect + fixed effect) of the interaction term combining mobility and agricultural hukou on health differences by interview locations in Models 1 to 7. (Words in the longitudinal axis represent the names of the survey regions).

Next, we considered whether the health effect from mobility on rural migrants’ health varied among different regions. We incorporated the random intercept and slope simultaneously into our seven previously constructed models, which allowed the coefficient of the interactive term combining agricultural hukou and migration to vary with different survey regions. Compared to the normal logit regression, the likelihood ratio test indicates that the random slope manifests significant disparities. In the next step, we determined the total effect (=random effect + fixed effect) of the interaction term combining mobility and agricultural hukou on health status in each interview location in the seven models. As shown in Table 12, with regard to the coefficient of the interaction term combining mobility and agricultural hukou, its absolute value and statistical significance both decreased as other characteristics gradually entered the function, which could verify that the positive health effect was from initial health rather than the migrant process. In Figure 1, the more visualized form, we can observe that the total health effect from the interaction term combining mobility and agricultural hukou turned from positive to negative in some interview locations when other related factors entered the function.

Before we began to explore the salmon bias hypothesis in China, we decided to use the Harmonized CHARLS (China Health and Retirement Longitudinal Study) data to further verify the healthy migrant hypothesis to improve the robustness check.

We used the dependent variable self-reported health in 2013 and 2015, the dummy variable agricultural hukou in 2013 (agricultural hukou = 1, others = 0), the dummy variable “work status” in 2013 (non-agricultural work = 1, others = 0 in 2012), and their interactive term as our independent variables of interest. Given the ordinal attributes of our dependent variables, we employed the ordered logit model to explore the health effect of mobility on migrant workers. In the Harmonized CHARLS data, there were two scales of self-reported health. Through our adjustment, we had four groups of self-reported health: One of the scales ranges from 1 for Poor to 5 for Excellent in 2013 and 2015, and the other scale ranges from 1 for Very Bad to 5 for Very Good in 2013 and 2015. As shown in Table 13, regardless of the method of defining self-reported health, the results are similar: The coefficient of agricultural hukou is negative and significant at the 1% level in the four models, which implies that rural residents’ health is generally worse than that of urban citizens. The coefficient of non-agricultural employment history is positive and significant at the 1% level. We are interested in the coefficient of the interaction term combining agricultural hukou and non-agricultural work experiences. In Model 1 and Model 3, using the dependent variable measuring health in 2013, the coefficient of the interaction term was positive and significant at the 5% level. When the dependent variable measured health status in 2015, the coefficient of both agricultural hukou and non-agricultural work experiences did not fluctuate conspicuously. In contrast, the coefficients of the interaction terms both became insignificant and even negative in Model 2, and their absolute values both obviously declined, which indicates that working in cities had a negative effect on the migrant workers. This finding is consistent with the previously generated results suggesting that the initial better health among migrant workers was due to self-selection. Healthier people tend to migrate for better remuneration, and the process of working outside their hukou-registered locale neutralizes their initial health advantage.

After testing the healthy migrant hypothesis in China, we chose samples who lived in rural areas in 2015 and whose work status in the 2013 survey questionnaire was non-agricultural work (i.e., engaged in non-agricultural work in 2012) to further explore the salmon bias hypothesis. First, we used two scales of the ordinal variable “self-reported health” as the dependent variable (one of the scales ranges from 1 for Poor to 5 for Excellent, the other scale ranges from 1 for Very Bad to 5 for Very Good), the dummy variable “work status” in 2013 (non-agricultural work = 1, others = 0 in 2012), the dummy variable “rural or urban residence” in 2015 (rural = 1, urban = 0), and their interactive term as our independent variables of interest. Considering the attributes of our ordinal dependent variable, we employed the ordered logit model. There is an overlap between agricultural hukou and living in a rural area. To assuage concerns about collinearity, we dropped the hukou category in our subsequent functional framework. As shown in Table 14, regardless of which approaches to measuring self-reported health we chose, the results were similar. The coefficient of non-agricultural work experience in 2012 was positive and significant at the 1% level in Models 1 and 2. In Models 3 and 4, when we added demographic and cognitive characteristics such as gender, age, and education level, the coefficient of non-agricultural work remained positive and significant at the 1% level. We are most interested in the coefficient of the interaction term combining living in rural areas in 2015 and non-agricultural work experience in 2012, which are insignificant and small in absolute value in all four models. Although we are aware of the initial better health condition among migrant workers when they start to migrate, the low statistical significance and low absolute value of the interaction term indicates that rural migrant workers who return to their hometowns tend to experience health deterioration.

Thanks to the copious amount of health indicators in the Harmonized CHARLS, we could utilize factor analysis with iterative common factor variance combined with oblique rotation, which permits the correlation among different factors to extract other health indicators. The factors obtained through oblique rotation can represent health conditions in different dimensions, which allowed us to vary the dependent variables to deploy a robustness check for the salmon bias hypothesis. However, the larger numbers of different health indicators represent worse health conditions in the Harmonized CHARLS. The independent variables have the opposite positive and negative coefficients when using symptoms or health behaviors to represent health compared with self-reported health. For instance, in the Harmonized CHARLS, the frequency of drinking in the previous year equals zero when the interviewee never drank in the previous year and equals eight when the interviewee drank more than twice per day; lower drinking frequency represents better health behavior. Since medical care utilization is constrained by the pecuniary budget and less affluent rural residents may endure disease or choose cheaper approaches to manage their health problems rather than going to hospital and seeking standard treatment, to assuage concerns of endogeneity, we excluded health care utilization or insurance and only included symptoms (for example, ADLs, IADLs, CESD10) and health behaviors (for example, drinking and smoking) to implement the factor analysis and further produce new factors representing the health condition in 2015.

We chose 20 variables in the Harmonized CHARLS to reflect the health condition in 2015: (1) Six-item summary of activities of daily living (ADL) containing bathing, dressing, eating, getting in/out of bed, using the toilet, and controlling urination (each item equals one if the interviewee had difficulty completing this item independently and otherwise equals zero); (2) five-item summary of instrumental activities of daily living (IADL) including whether the interviewee had difficulty managing money, taking medications, shopping for groceries, preparing meals, and making phone calls; (3) seven-item summary of any difficulty with mobility activities, including walking 100 m, climbing several flights of stairs, getting up from a chair, stooping, kneeling or crouching, extending arms up, lifting 5 kg, and picking up a small coin; (4) CESD10 ranging from 0 to 30 with higher scores indicating that the respondent felt more negative during the past week; (5)–(17) the respondent’s answer to the question regarding whether a doctor had told the respondent that he or she had a specific condition, including high blood pressure; diabetes or high blood sugar; cancer or a malignant tumor; chronic lung disease; heart attack, coronary heart disease, angina, congestive heart failure, or other heart problems; stroke; emotional, nervous, or psychiatric problems; arthritis; dyslipidemia; liver disease; kidney disease; stomach or other digestive disease; and asthma; (18) the respondent’s response to the question regarding whether a doctor had told the respondent that he or she had a memory-related condition; (19) frequency of drinking behavior during the last year; and (20) current smoking habit.

The Kaiser-Meyer-Olkin measure of sampling adequacy in Appendix C shows that the KMO value of the majority of the variables is more than 0.7 and the overall KMO value is 0.7398, which indicates that the factor analysis is appropriate.

This large number of variables may reflect fewer potential dimensions. We employed the principal factor method combined with iterated communalities, and through oblique rotation, we extracted four factors to represent health in different dimensions. According to the rotation result, the extracted four factors can represent daily activities, internal disease, organ disease, and unhealthy behaviors. Daily activities include ADL, IADL, mobility difficulties, CESD10; internal disease include high blood pressure, memory-related conditions, stroke, diabetes, cancer, psychiatric problems, heart problems, and dyslipidemia; organ disease includes lung, digestive, asthma, arthritis, liver, and kidney problems; and unhealthy behavior includes drinking and smoking habits.

As shown in Table 15, we employ Ordinary Least Square (OLS) to examine the salmon bias hypothesis. When the dependent variables are daily activities or organ diseases, the results are consistent with our previously observed results. Regardless of whether the demographic and cognitive control variables are included, healthier people tended to perform non-agricultural work in 2012. In comparison, the coefficient of our interaction term of greatest interest combining non-agricultural work experience in 2012 and living in a rural area in 2015 declined in both absolute value and statistical significance, which implies that the returnees often had declining health. Interestingly, when the dependent variables were internal disease or unhealthy behavior, some fluctuations emerged. The results of the OLS model suggest that individuals living in rural areas in 2015 or working in non-agricultural departments in 2012 had fewer internal diseases, while those who lived in rural areas in 2015 and simultaneously had non-agricultural work experiences tended to suffer more internal diseases compared to others. Given the initial better health condition among rural migrant workers, this result strongly supports the salmon bias hypothesis. Those who choose to return to their rural hometown often lose their health advantages, and a non-agricultural employment history can produce chronic diseases among them via poor working environments. According to the regression results of the last two OLS function frameworks, the returnees seem to have less unhealthy behavior compared to their peers who remained in destination cities, which may reveal a possible mechanism of health deterioration by which living and working in cities without urban hukou could lead to alcohol drinking and smoking. These unhealthy behaviors may temporarily alleviate socioeconomic pressure and depression, but living and working in cities without urban hukou may also lead to drinking alcohol and smoking.

## 4. Discussion and Conclusions

This paper examined the “healthy migrant hypothesis” and the “salmon bias hypothesis” in China. Our empirical evidence supports both hypotheses in the Chinese context. In the ordered logit model, when we included only mobility, agricultural hukou, and their interactive term, the coefficient of the interaction term was positive and significant at 1% level. When we gradually added other demographic, cognitive, and socioeconomic characteristics, this positive effect disappeared in both absolute value and statistical significance. When we included the random intercept and random slope in the mixed-effect logit model, which allowed the intercept and the slope of the interaction term combining agricultural hukou to shift when different subgroups of the sample changed, this phenomenon persisted, which suggests that the health priorities among rural migrant workers can be attributed to their previous self-selection rather than the migrant effect. People endowed with initial better heath are more likely to migrate to cities seeking economic opportunities. Under the draconian hukou system, their career choices, access to local medical care, and opportunities for public services are limited in the host cities, and they may suffer discrimination from native citizens and mistreatment from their employers. Consequently, their initial health advantage gradually disappears. Because China’s New Rural Cooperative Medical System is only valid in hukou-registered locations, rural migrant workers tend to return to their hometown after their health deteriorates.

The difficulties of rural migrant workers can be blamed in part on broader conditions, such as the inability to obtain a strong connection between destination cities’ public services and local hukou; the presence at the nadir of career ladders caused by inadequate knowledge and the hukou system; dangerous and even polluted working environments; and crowded and dirty living conditions. After migrant workers experience deteriorating health, returning to their rural hometowns seems to be their best choice. According to our empirical outcomes, the returnees often had declining health. This is a serious problem in China. As the “healthy migrant” and “salmon bias” have consistent effects, the burden on the new rural cooperative medical system is continually increased, and the already widening gap of population health between urban and rural China further enlarges. The conclusions of this article provide important policy implications. Rural migrant workers must adapt if they are to survive in destination cities, and governments can help them to do so, such as by loosening the linkage between local hukou and medical care and providing more public services (especially low-rent housing) and occupation choices. Eliminating the problem altogether will be impossible, but considering the vitally important role rural migrant workers play in long-term development and the importance of promoting people’s happiness and perceptions of equality perception, it is time to help them overcome various obstacles in cities rather than allowing hukou to colonize its role of continuously signaling permission.

This article makes the following important contributions. (1) China has experienced the largest domestic migration process in human history. Empirical testing of the relationship between rural-urban migration and health in China can help people to more accurately understand the relationship between population migration and health. (2) We present some innovations in the research methods. Considering that rural and urban dualization formed under the hukou system, we utilized two dimensions—agricultural hukou and migrant status—to employ difference-in-difference (DID) to focus on the interaction term combining agricultural hukou and migration. Furthermore, we used propensity score matching-difference in difference (PSM-DID) to find a more comparable group and overcome some of the defects of DID to examine the healthy migrant hypothesis. Moreover, considering that interviewees in different regions may have different criteria for health conditions, we incorporated the random intercept of each survey site and the random slope of the interactive term combining agricultural hukou and migration into the mixed-effect logit regression model. (3) Existing articles often focus on a single independent variable and usually use one micro database. Based on the existing research, we explored the “healthy migrant hypothesis” and the “salmon bias hypothesis” under Chinese household registration systems by focusing on the interaction term combining agricultural hukou and migrant status, previous non-agricultural working experiences and subsequent residence in rural areas. We also utilized two Chinese micro-databases to make our conclusions more cogent. (4) Urbanization and its concomitant rural-urban migration are objective processes, and many developing countries are experiencing an urbanization process similar to that in China. In this regard, the conclusions of this paper on rural-urban migration and health can provide more general value.

There are some limitations of our study. For example, because our focus was on the interaction term, the Heckman two-step method and the entropy balanced matching method were not used. Furthermore, the mental health of Chinese migrant workers was not used as a dependent variable to observe whether it is applicable to the healthy migrant hypothesis and the salmon bias hypothesis. We leave these aspects for future research.

## Figures and Tables

**Table 1:** Descriptive statistics of key variables.

VarName.	Obs ^1^	Mean	SD ^2^	Min	Median	Max
Self-reported health	10,961	3.61	1.075	1	4	5
Agricultural hukou	10,924	0.10	0.298	0	0	1
Age	10,956	0.57	0.496	0	1	1
Female	10,968	50.40	16.898	18	50	95
Single	10,968	0.53	0.499	0	1	1
Education	10,968	0.22	0.413	0	0	1
Household income per capita	10,939	4.87	3.113	1	4	13
Possess private car or not	10,037	26,700.90	1.56 × 10^5^	0	12,500	9.08 × 10^6^
Socioeconomic status compared with peers	10,955	0.17	0.376	0	0	1
Self-rated family’s financial situation	10,904	2.65	0.717	1	3	5
Autonomy in determining the way of working	10,903	1.71	0.549	1	2	3
Whether the company is within the system	3975	2.77	0.958	1	3	4
Whether the company is a state-owned enterprise	3831	0.55	0.498	0	1	1
Have medical insurance or not	2307	0.36	0.480	0	0	1
Whether current work includes a signed labor contract with the employer	10,917	0.91	0.283	0	1	1
Fluency level of speaking Mandarin	3711	0.45	0.498	0	0	1
Frequency of participation in cultural events	10,960	3.16	1.200	1	3	5
Frequency of depression	10,876	1.49	0.817	1	1	5
Social equality perception	10,942	3.84	0.924	1	4	5
Whether a CCP ^3^ member or not	10,904	3.20	1.005	1	3	5

^1^ Observation (Obs); ^2^ Standard Deviation (SD); ^3^ Chinese Communist Party (CCP).

**Table 2:** Descriptive statistics of key variables in Harmonized CHARLS.

VarName	Obs ^1^	Mean	SD ^2^	Min	Median	Max
Wave2 self-reported health	17,591	2.17	0.927	1	2	5
Wave2 self-reported health alternative	17,603	3.03	0.957	1	3	5
Wave4 self-reported health	19,701	2.20	0.948	1	2	5
Wave4 self-reported health alternative	19,698	3.11	0.990	1	3	5
W2agricultural hukou	18,551	0.76	0.424	0	1	1
W2non-agricultural work	25,504	0.11	0.311	0	0	1
W4liverural	21,097	0.59	0.491	0	1	1
Female	25,493	0.52	0.500	0	1	1
Education	25,419	3.47	1.929	1	4	10
W4age	21,054	59.13	10.751	18	59	115
Frequency of drinking behavior	20,848	1.24	2.317	0	0	8
Smoking habit	20,920	0.28	0.449	0	0	1
Six-item summary of ADL ^3^	20,195	0.41	1.047	0	0	6.00
Five-item summary of IADL ^4^	18,386	0.46	1.012	0	0	5
Seven-item summary of any difficulty with mobility activities	20,197	1.38	1.685	0	1	7
CESD10 ^5^	19,611	7.82	6.331	0	6	30
High blood pressure	17,237	0.36	0.481	0	0	1
Diabetes or high blood sugar	16,944	0.11	0.314	0	0	1
Cancer or a malignant tumour	17,018	0.02	0.143	0	0	1
Chronic lung disease	17,076	0.16	0.367	0	0	1
Heart problems	17,047	0.20	0.397	0	0	1
Stroke	17,083	0.04	0.204	0	0	1
Emotional, nervous, or psychiatric problems	17,043	0.03	0.162	0	0	1
Arthritis	17,200	0.46	0.499	0	0	1
Dyslipidaemia	16,700	0.19	0.396	0	0	1
Liver disease	17,010	0.07	0.262	0	0	1
Kidney disease	17,024	0.11	0.316	0	0	1
Stomach or other digestive disease	17,191	0.34	0.473	0	0	1
Asthma	17,053	0.06	0.246	0	0	1
Memory-related condition	20,770	0.03	0.170	0	0	1

^1^ Observation (Obs); ^2^ Standard Deviation (SD); ^3^ Activities of daily living (ADL); ^4^ Instrumental activities of daily living (IADL); ^5^ 10-item Center for the Epidemiological Studies of Depression (CESD10).

**Table 3:** The Difference-in-Difference construction.

	Migrant	Non-Migrant
Non-agricultural hukou	E(SRH|*mobility* = 1, *agrihk* = 0)	E(SRH|*mobility* = 0, *agrihk* = 0)
Agricultural hukou	E(SRH|*mobility* = 1, *agrihk* = 1)	E(SRH|*mobility* = 0, *agrihk* =1 )

SRH: Self-rated health.

**Table 4:** The Difference-in-Difference construction and the numeric results.

	Migrant	Non-Migrant	Difference
Non-agricultural hukou	$\alpha_{0}+\alpha_{1}=3.853_{}$	$\alpha_{0}=3.662^{}$	$\alpha_{1}=0.192^{\;***}$
Agricultural hukou	$\alpha_{0}+\alpha_{1}+\alpha_{2}+\alpha_{3}=3.991^{}$	$\alpha_{0}+\alpha_{2}=3.507_{}$	$\alpha_{1}+\alpha_{3}=0.484^{\;***}$
Difference	$\alpha_{2}+\alpha_{3}=0.138^{}$	$\alpha_{2}=0.155^{}$	$\alpha_{3}=0.292^{\;***}$

*** *p* < 0.001, ** *p* < 0.01, * *p* < 0.005, ^+^ *p* < 0.1.

**Table 5:** The Difference-in -Difference (DID) construction and the numeric results.

	Model (1)	Model (2)
*agrihk*	−0.155 ***	−0.175 ***
*-*	(0.022)	(0.020)
*mobility*	0.192 ***	0.035
*-*	(0.043)	(0.041)
*_diff*	0.292 ***	0.151 *
*-*	(0.062)	(0.059)
*age*	-	−0.024 ***
*-*	-	(0.001)
*female*	-	−0.149 ***
*-*	-	(0.019)
*single*	-	−0.063 **
*-*	-	(0.023)
Constant	3.662 ***	5.011 ***
-	(0.016)	(0.034)
Observations	10,911	10,911
R-squared	0.014	0.159

Standard errors in parentheses; *** *p* < 0.001, ** *p* < 0.01, * *p* < 0.05.

**Table 6:** The KMO (Kaiser-Meyer-Olkin) value of variables.

Variable	KMO
household income per capita	0.7762
possess private car or not	0.8050
socioeconomic status compared with peers	0.6660
self-rated family’s financial situation	0.6695
autonomy in determining the way of working	0.8025
whether the company is within the system	0.5805
whether the company is a state-owned enterprise	0.5877
have medical insurance or not	0.6961
whether the current work involves a signed labor contract with the employer	0.6260
fluency level of speaking Mandarin	0.7343
frequency of participation in cultural events	0.7491
frequency of depression	0.6464
social equality perception	0.6924
whether a CCP member or not	0.8119
overall	0.6775

**Table 7:** The results of the PSM (propensity score matching) model.

	Model (1)	Model (2)	Model (3)
	psm1:1	psmkernel	psmllr
Variables	SRH	SRH	SRH
Average treatment effect on treated group	0.0251	−0.013	−0.005
-	(0.071)	(0.051)	(0.071)
T-stat	0.350	−0.250	−0.080
Observations	1966	1966	1966
R-squared	0.068	0.068	0.068

SRH: Self-rated health.

**Table 8:** Result of PSM-DID (Propensity score matching combined Difference-in-Difference) model.

Variables	PSM-DID
*agrihk*	0.000
	(0.000)
*mobility*	−0.022
	(0.045)
*_diff*	0.000
	(0.000)
Constant	4.084 ***
	(0.032)
Observations	1260
R-squared	0.000

Standard errors in parentheses; *** *p* < 0.001, ** *p* < 0.01, * *p* < 0.05, ^+^ *p* < 0.1.

**Table 9:** The result of *t*-test.

Variable	Diff	|t|	*p* Value
*SRH* (*Self-rated health)*	−0.022	0.480	0.629
*age*	−0.340	0.590	0.557
*female*	−0.008	0.300	0.764
*single*	−0.009	0.340	0.730
*education*	−0.046	0.270	0.784
*subjective*	−0.035	0.760	0.448
*career*	−0.011	0.250	0.800
*objective*	−0.002	0.040	0.966

**Table 10:** The result of ordered logit regression.

	Model (1)	Model (2)	Model (3)	Model (4)	Model (5)	Model (6)	Model (7)
Variables	SRH	SRH	SRH	SRH	SRH	SRH	SRH
*mobility*	0.308 ***	0.039	−0.000	−0.111	−0.089	−0.111	−0.111
	(0.082)	(0.083)	(0.084)	(0.144)	(0.144)	(0.143)	(0.145)
*agrihk*	−0.227 ***	−0.297 ***	−0.147 ***	0.044	0.104	0.163	0.056
	(0.037)	(0.037)	(0.043)	(0.113)	(0.114)	(0.114)	(0.116)
mobility*agrihk	0.531 ***	0.327 **	0.333 **	0.256	0.126	0.146	0.252
	(0.117)	(0.119)	(0.119)	(0.223)	(0.222)	(0.222)	(0.224)
*age*		−0.046 ***	−0.041 ***	−0.042 ***	−0.040 ***	−0.040 ***	−0.042 ***
		(0.001)	(0.001)	(0.005)	(0.005)	(0.005)	(0.005)
*female*		−0.326 ***	−0.288 ***	−0.240 *	−0.239 *	−0.249 *	−0.242 *
		(0.040)	(0.040)	(0.097)	(0.097)	(0.097)	(0.097)
*single*		−0.177 **	−0.190 **	−0.252 ^+^	−0.285 *	−0.246 ^+^	−0.247 ^+^
		(0.064)	(0.064)	(0.142)	(0.142)	(0.142)	(0.143)
female*single		0.191 *	0.176 *	0.168	0.145	0.150	0.167
		(0.087)	(0.087)	(0.210)	(0.209)	(0.209)	(0.210)
*education*			0.053 ***	−0.019	0.015	−0.016	−0.022
			(0.007)	(0.016)	(0.016)	(0.017)	(0.018)
*subjective*				0.370 ***			0.354 ***
				(0.055)			(0.067)
*career*					0.031		0.004
					(0.056)		(0.060)
*objective*						0.292 ***	0.038
						(0.072)	(0.092)
Observations	10,911	10,911	10,884	1964	1964	1964	1964

SRH: Self-rated health. Standard errors in parentheses; *** *p* < 0.001, ** *p* < 0.01, * *p* < 0.05, ^+^ *p* < 0.1. “mobility*agrihk” represents the interactive term combined “mobility” and “agricultural hukou”; “female*single” represents the interactive term combined “female” and “single”. Similarly hereinafter.

**Table 11:** The result of mixed-effect logit regression with random intercept.

Variables	Model (1)	Model (2)	Model (3)	Model (4)	Model (5)	Model (6)	Model (7)
*mobility*	0.471 ***	0.226 *	0.190 ^+^	0.220	0.232	0.254	0.205
	(0.103)	(0.110)	(0.111)	(0.209)	(0.209)	(0.208)	(0.210)
*agrihk*	−0.269 ***	−0.305 ***	−0.128 *	0.205	0.236	0.293 ^+^	0.184
	(0.047)	(0.051)	(0.055)	(0.159)	(0.160)	(0.160)	(0.163)
mobility*agrihk	0.401 **	0.115	0.157	−0.015	−0.110	−0.118	0.001
	(0.146)	(0.154)	(0.156)	(0.322)	(0.320)	(0.320)	(0.323)
*age*		−0.046 ***	−0.040 ***	−0.045 ***	−0.043 ***	−0.043 ***	−0.045 ***
		(0.001)	(0.002)	(0.006)	(0.006)	(0.006)	(0.006)
*female*		−0.365 ***	−0.303 ***	−0.221 ^+^	−0.220 ^+^	−0.217 ^+^	−0.222 ^+^
		(0.048)	(0.048)	(0.131)	(0.130)	(0.130)	(0.131)
*single*		−0.110	−0.123	−0.137	−0.198	−0.138	−0.154
		(0.083)	(0.083)	(0.208)	(0.208)	(0.208)	(0.210)
female*single		0.153	0.142	−0.097	−0.115	−0.127	−0.090
		(0.110)	(0.111)	(0.297)	(0.294)	(0.294)	(0.297)
*education*			0.078 ***	0.023	0.066 **	0.037	0.030
			(0.010)	(0.023)	(0.023)	(0.024)	(0.025)
*subjective*				0.368 ***			0.389 ***
				(0.075)			(0.092)
*career*					−0.036		−0.035
					(0.077)		(0.085)
*objective*						0.231 *	−0.048
						(0.100)	(0.134)
Constant	0.530 ***	3.142 ***	2.349 ***	3.067 ***	2.647 ***	2.840 ***	3.021 ***
	(0.077)	(0.117)	(0.153)	(0.404)	(0.399)	(0.398)	(0.411)
Observations	10,911	10,911	10,884	1964	1964	1964	1964

Standard errors in parentheses; *** *p* < 0.001, ** *p* < 0.01, * *p* < 0.05, ^+^ *p* < 0.1.

**Table 12:** The result of mixed-effect logit regression with a random slope.

Variables	Model (1)	Model (2)	Model (3)	Model (4)	Model (5)	Model (6)	Model (7)
*mobility*	0.367 ***	0.120	0.069	0.021	0.027	0.034	0.018
	(0.100)	(0.107)	(0.108)	(0.202)	(0.202)	(0.201)	(0.204)
*agrihk*	−0.192 ***	−0.252 ***	−0.061	0.199	0.244	0.295+	0.155
	(0.042)	(0.044)	(0.051)	(0.150)	(0.149)	(0.151)	(0.154)
mobility*agrihk	0.406 **	0.151	0.174	0.179	0.023	0.042	0.185
	(0.147)	(0.159)	(0.161)	(0.393)	(0.378)	(0.385)	(0.390)
*age*		−0.045 ***	−0.040 ***	−0.046 ***	−0.043 ***	−0.044 ***	−0.046 ***
		(0.001)	(0.002)	(0.006)	(0.006)	(0.006)	(0.006)
*single*		−0.133	−0.148 ^+^	−0.135	−0.206	−0.162	−0.156
		(0.081)	(0.081)	(0.203)	(0.202)	(0.203)	(0.205)
*female*		−0.348 ***	−0.291 ***	−0.232 ^+^	−0.232 ^+^	−0.232 ^+^	−0.231 ^+^
		(0.047)	(0.048)	(0.128)	(0.127)	(0.127)	(0.128)
single*female		0.160	0.151	−0.124	−0.141	−0.152	−0.114
		(0.109)	(0.110)	(0.290)	(0.287)	(0.287)	(0.291)
*education*			0.071 ***	0.013	0.054 *	0.031	0.024
			(0.009)	(0.022)	(0.022)	(0.023)	(0.024)
*subjective*				0.374 ***			0.428 ***
				(0.073)			(0.088)
*career*					−0.026		−0.007
					(0.074)		(0.081)
*objective*						0.174+	−0.133
						(0.096)	(0.125)
Constant	0.477 ***	3.079 ***	2.340 ***	3.138 ***	2.739 ***	2.895 ***	3.088 ***
	(0.032)	(0.089)	(0.131)	(0.382)	(0.377)	(0.376)	(0.390)
Observations	10,911	10,911	10,884	1964	1964	1964	1964

Standard errors in parentheses; *** *p* < 0.001, ** *p* < 0.01, * *p* < 0.05, ^+^ *p* < 0.1

**Table 13:** Using the China Health and Retirement Longitudinal Survey (CHARLS) to employ a robustness check for the healthy migrant hypothesis.

	Model (1)	Model (2)	Model (3)	Model (4)
Variables	w2SRH ^1^	w4SRH	w2SRHal	w4SRHal
w2agrihk	−0.352 ***	−0.333 ***	−0.321 ***	−0.319 ***
	(0.038)	(0.042)	(0.038)	(0.042)
w2nonagri	0.594 ***	0.690 ***	0.652 ***	0.617 ***
	(0.068)	(0.078)	(0.068)	(0.077)
w2agrihk*w2nonagri	0.175 *	−0.010	0.183 *	0.011
	(0.084)	(0.093)	(0.083)	(0.093)
cut1	−1.469 ***	−1.436 ***	−3.068 ***	−3.100 ***
	(0.036)	(0.040)	(0.046)	(0.051)
cut2	0.918 ***	1.029 ***	−1.277 ***	−1.343 ***
	(0.035)	(0.039)	(0.035)	(0.040)
cut3	1.917 ***	1.857 ***	1.071 ***	1.055 ***
	(0.038)	(0.042)	(0.035)	(0.039)
cut4	4.156 ***	4.245 ***	2.198 ***	1.942 ***
	(0.071)	(0.080)	(0.040)	(0.042)
Observations	17,553	15,293	17,566	15,290

^1^ Self-rated health (SRH). Standard errors in parentheses; *** *p* < 0.001, ** *p* < 0.01, * *p* < 0.05, ^+^ *p* < 0.1. “w2agrihk*w2nonagri” represents the interactive term combined “agricultural hukou in 2013” and “non-agrcultural work in 2013”; “cut1,..., cut4” is the estimated value of cutoff points. Similarly hereinafter.

**Table 14:** Using self-reported health as the dependent variable to explore the salmon bias hypothesis.

	Model (1)	Model (2)	Model (3)	Model (4)
Variables	w4SRH	w4SRHal	w4SRH	w4SRHal
w4 liverural	−0.319 ***	−0.299 ***	−0.287 ***	−0.262 ***
	(0.030)	(0.029)	(0.031)	(0.030)
w2nonagri	0.487 ***	0.438 ***	0.298 ***	0.254 ***
	(0.058)	(0.058)	(0.059)	(0.059)
w4 liverural*w2nonagri	0.073	0.079	0.065	0.066
	(0.084)	(0.084)	(0.084)	(0.084)
female			−0.266 ***	−0.274 ***
			(0.029)	(0.029)
education			0.050 ***	0.051 ***
			(0.008)	(0.008)
w4age			−0.021 ***	−0.020 ***
			(0.001)	(0.001)
cut1	−1.497 ***	−3.137 ***	−2.747 ***	−4.290 ***
	(0.026)	(0.039)	(0.105)	(0.108)
cut2	0.933 ***	−1.388 ***	−0.273 **	−2.523 ***
	(0.025)	(0.026)	(0.103)	(0.103)
cut3	1.749 ***	0.965 ***	0.553 ***	−0.131
	(0.028)	(0.024)	(0.103)	(0.102)
cut4	4.137 ***	1.850 ***	2.949 ***	0.765 ***
	(0.063)	(0.028)	(0.117)	(0.102)
Observations	19,701	19,698	19,649	19,646

SRH: Self-rated health. Standard errors in parentheses; *** *p* < 0.001, ** *p* < 0.01, * *p* < 0.05, ^+^ *p* < 0.1. “w4liverural*w2nonagri” represents the interaction term combined “residing in rural areas in 2015” and “non-agrcultural work in 2012”; “cut1,..., cut4” is the estimated value of cutoff points. Similarly hereinafter.

**Table 15:** Using different health factors as dependent variables to employ a robustness check.

	Model (1)	Model (2)	Model (3)	Model (4)	Model (5)	Model (6)	Model (7)	Model (8)
VARIABLES	daily	daily	inside	inside	organ	organ	behavior	behavior
w4 liverural	0.146 ***	0.117 ***	−0.171 ***	−0.125 ***	0.062 ***	0.052 **	0.040 **	0.006
	(0.018)	(0.017)	(0.015)	(0.016)	(0.016)	(0.016)	(0.014)	(0.012)
w2nonagri	−0.443 ***	−0.158 ***	−0.291 ***	−0.168 ***	−0.251 ***	−0.141 ***	0.259 ***	0.062 **
	(0.032)	(0.031)	(0.028)	(0.028)	(0.029)	(0.029)	(0.025)	(0.021)
w4 liverural*w2nonagri	−0.025	−0.039	0.071 ^+^	0.062	−0.009	−0.030	0.081 *	0.028
	(0.045)	(0.043)	(0.039)	(0.038)	(0.040)	(0.040)	(0.036)	(0.029)
female		0.284 ***		0.197 ***		−0.022		−0.864 ***
		(0.016)		(0.014)		(0.015)		(0.011)
education		−0.066 ***		0.026 ***		−0.025 ***		−0.008 **
		(0.004)		(0.004)		(0.004)		(0.003)
w4age		0.021 ***		0.015 ***		0.011 ***		−0.005 ***
		(0.001)		(0.001)		(0.001)		(0.001)
Constant	−0.029 *	−1.224 ***	0.145 ***	−0.989 ***	−0.003	−0.605 ***	−0.068 ***	0.769 ***
	(0.014)	(0.064)	(0.012)	(0.058)	(0.013)	(0.060)	(0.011)	(0.043)
Observations	12,986	12,983	12,986	12,983	12,986	12,983	12,986	12,983
R-squared	0.040	0.142	0.020	0.056	0.015	0.039	0.022	0.372

Standard errors in parentheses; *** *p* < 0.001, ** *p* < 0.01, * *p* < 0.05, ^+^ *p* < 0.1.

## Appendix C

**Table A1:** KMO (Kaiser-Meyer-Olkin) value of variables selected in Harmonized CHARLS to employ factor analysis.

Variables	KMO
six-item summary of ADL ^1^	0.7213
five-item summary of IADL ^2^	0.8695
seven-item summary of any difficulty with mobility activities	0.7234
CESD10 ^3^	0.8568
high blood pressure	0.7421
diabetes or high blood sugar	0.7261
cancer or a malignant tumour	0.7984
chronic lung disease	0.6260
heart problems	0.7989
stroke	0.8185
emotional, nervous, or psychiatric problems	0.7733
arthritis	0.8240
dyslipidaemia	0.6949
liver disease	0.7422
kidney disease	0.8092
stomach or other digestive diseases	0.7631
asthma	0.5950
memory-related condition	0.8188
frequency of drinking behaviour	0.6025
smoking habit	0.6041
overall	0.7398

^1^ Activities of daily living (ADL); ^2^ Instrumental activities of daily living (IADL); ^3^ 10-item Center for the Epidemiological Studies of Depression (CESD10).
